# Risk factors for developing acute gastrointestinal, skin or respiratory infections following obstacle and mud run participation, the Netherlands, 2017

**DOI:** 10.2807/1560-7917.ES.2019.24.40.1900088

**Published:** 2019-10-03

**Authors:** Elisabeth M den Boogert, Danielle M Oorsprong, Ewout B Fanoy, Alexander CAP Leenders, Alma Tostmann, Adriana SG van Dam

**Affiliations:** 1 Department of Infectious Disease Control, Municipal Health Service Hart voor Brabant, ‘s-Hertogenbosch, the Netherlands; 2 Department of Infectious Disease Control, Public Health Service Rotterdam-Rijnmond, Rotterdam, the Netherlands; 3 Centre for Infectious Diseases, Epidemiology and Surveillance, National Institute for Public Health and the Environment (RIVM), Bilthoven, the Netherlands; 4 Department of Medical Microbiology and Infection Control, Jeroen Bosch Hospital, ‘s-Hertogenbosch, the Netherlands; 5 Academic Public Health Initiative AMPHI, Department of Primary and Community Care, Radboud Centre for Infectious Diseases, Nijmegen, the Netherlands

**Keywords:** gastrointestinal disease, respiratory infections, hygiene, infection control, mass gatherings, epidemiology

## Abstract

**Background:**

In the Netherlands, obstacle, mud and survival runs are increasingly popular. Although outbreaks of gastroenteritis have been reported following these events, associated health risks have not been systematically assessed.

**Aim:**

To investigate the incidence of acute gastrointestinal infections (AGI), skin infections (SI) and respiratory infections (RI) among obstacle run participants, as well as risk factors.

**Methods:**

Between April and October 2017, we conducted a retrospective cohort study among 2,900 participants of 17 obstacle runs in the Netherlands. Demographic, symptomatic and behavioural data were collected from participants via an online questionnaire 1 week after participation in an obstacle run. Stool specimens were obtained from respondents for microbiological tests. Adjusted relative risks (aRR) and 95% confidence intervals (CI) using multilevel binomial regression analysis were calculated.

**Results:**

Of 2,646 respondents (median age: 33 years; 53% male), 76 had AGI after the obstacle run; ingesting mud was associated with AGI (aRR: 1.7; 95% CI: 1.2–4.9) and 38 respondents had AGI during or in the week before the obstacle run. Overall, 103 respondents reported SI and 163 RI. Rinsing off in a hot tub was associated with SI (aRR: 2.2; 95% CI: 1.7–2.8). Of 111 stool specimens, 13 tested positive for six different pathogens. No clusters were found.

**Conclusion:**

The reported incidence of AGI, SI and RI was low. Risk of these infections could be decreased by informing participants on preventive measures, e.g. showering vs rinsing in the hot tub, avoiding ingesting mud and not participating with symptoms of AGI.

## Introduction

In the Netherlands, obstacle, mud and survival runs (for the purposes of this paper, collectively referred to as ‘obstacle runs’) are increasingly popular. Initially, trained or professional runners were the main participants in these races, but the sport has developed into a fun activity for friends and families. There is a growing number of participants each year (13,000 in 2012 to > 250,000 in 2017 [1]). The minimum age for participation varies between obstacle runs; it is usually based on the run’s distance and can be as young as 5 years old [2]. In 2017, over 150 obstacle runs were organised in the Netherlands [3]. Obstacle runs are races in which participants encounter different manufactured obstacles while running around a predefined course [4]. A mud run is basically the same, but intentionally features more mud [4]. Survival runs are a combination of an obstacle run and an endurance event; these require more technique and training than obstacle runs and are often non-commercial compared to obstacle races and mud runs [5,6].

As participants of obstacle runs are required to run, crawl or swim through untreated water and mud, risk of injury and infectious diseases such as acute gastrointestinal infection (AGI), respiratory infection (RI) and skin infection (SI) can be more prevalent in these races compared to more conventional running races. Since 2010, there have been multiple reports of AGI outbreaks following obstacle runs, open water swimming events and mountain biking events, and the ingestion of mud or water during these races was associated with the infections [7-14]. In Belgium and the Netherlands, several cases of leptospirosis were reported in 2015 after participation in an obstacle run and in the Netherlands, one case of tularaemia was linked to an obstacle run [15-17]. However, these reports do not provide information on the infectious disease risks and potential risk factors associated with obstacle run participation in general.

Although there are publications about outbreaks following obstacle runs, a more systematic approach to identify the events’ potential risk factors is lacking. Research on the potential health risks of obstacle runs is therefore warranted, and outcomes could potentially support recommendations that may help to further improve safety and preventive measures at these events.

This study investigated potential risk factors for developing AGI, RI or SI—such as accidentally swallowing mud/water, time between finish and rinsing off, or type of clothes worn—following participation in obstacle runs in the Netherlands. With the results, we aim to develop evidence-based preventative recommendations for organisers and participants of obstacle runs.

## Methods

### Study design

A retrospective cohort study was performed in four of 12 provinces in the Netherlands (Zuid-Holland, Noord-Brabant, Limburg and Gelderland) among participants of 17 obstacle runs that took place between April and October 2017. Obstacle runs were selected based on small (n < 1,000) and large (n > 1,000) numbers of participants to include both professional and voluntary organisations.

### Study population

The study population was defined as all participants that took part in at least one of the 17 selected obstacle runs. Organisers of each obstacle run were contacted before the race and given information on the study. They were asked to send a message to all participants. The message contained a link to an online questionnaire and was sent via email or posted to social media or the obstacle run’s website 7–10 days after the event. For organisers, sending the message to participants was considered consent for participation in the study; for participants, starting the questionnaire was considered giving consent.

### Case definitions

Case definitions were based on guidelines from the National Coordination for Communicable Diseases Control in the Netherlands [18,19] and were defined by a medical doctor and an infectious disease epidemiologist.

Box **Case definitions for acute gastrointestinal, respiratory and skin infections, the Netherlands, 2017**

**AGI** was defined as the development of any diarrhoea and/or vomiting within 14 days after the run.

**SI** was defined as the development of red bumps on the skin or other skin abnormalities within 14 days after the run.

**RI** was defined as the development of a cold, sore throat or cough within 14 days after the run.

**Injuries** were defined as muscle or joint injuries contracted during the run.

**Wound** was defined as a wound (cut or abrasion) contracted during the run.

AGI: acute gastrointestinal infection; RI: respiratory infection; SI: skin infection.

### Data collection

#### Epidemiological

Questionnaire data were collected via Collector innovative surveys [20]. The questionnaire included questions related to: (i) demographic characteristics; (ii) run-specific information including distance, time started and duration; (iii) health complaints experienced before, during and after the obstacle run, e.g. vomiting, diarrhoea, headache, injuries and wounds; and (iv) potential risk factors for infectious diseases, e.g. swallowing water/mud, type of clothing worn, food and drinks consumed, time between completion of the obstacle run and showering, chronic diseases (e.g. hay fever or other self-defined allergies, diabetes, immune disorders) and medications taken (e.g. antacids, antibiotics). Organisers could also add their own questions to get feedback regarding the organisation of the obstacle run.

Participants had ca 1 week to complete the questionnaire. Organisers of 12 of the obstacle runs also sent a reminder email 3–8 days after the first invitation or posted a reminder on social media. An example of the questionnaire can be seen in Supplement S1.

#### Microbiological

Respondents with known contact details who indicated that they had symptoms of AGI before, during or after the run were asked if they would be willing to collect a stool specimen and send it to Jeroen Bosch Hospital’s microbiological laboratory for analysis. If they accepted, a stool sample taking kit was sent to their home address. Where possible, 10 respondents reporting AGI symptoms per obstacle run were included for stool testing within 3 weeks after the run; if less than 10 respondents reported symptoms of AGI, respondents who did not report symptoms of AGI were invited to provide a stool sample. This decision was made on the basis that an asymptomatic individual infected with a pathogen could still be a potential risk for onward transmission.

Stool specimens were tested for *Salmonella* species*, Shigella* spp.*, Campylobacter* spp., Shigatoxin producing *Escherichia coli* (STEC), noro- and sapovirus, *Entamoeba histolytitica, Cryptosporidium parvum/hominis* and *Giardia lamblia*, all by reverse transcriptase-PCR (RT-PCR). Specimens were also tested for rota- and adenovirus infections with a qualitative immunochromatographic test. STEC-positive specimens were further tested by RT-PCR to determine whether the strain belonged to the subgroup of enterohaemorrhagic *Escherichia coli* (EHEC).

#### Environmental

A checklist was developed to identify potential environmental health hazards at an obstacle run. During each of the 17 obstacle runs we visually inspected the trails for animal faeces and asked the organisers whether the water had been officially approved by local authorities for swimming, according to the items provided on the checklist. We assessed the hygienic standards of facilities—e.g. toilets, showers and food trucks—in the event area and also took water samples at two or three random points along the runs. These were collected so that samples would be rapidly available for analysis in the case of an outbreak. They were not collected for comparison between obstacle runs and were stored following collection.

### Outbreak definition

An outbreak was defined as multiple participants of an obstacle run reporting the same clinical symptoms following participation and/or submitting stool specimens that tested positive for the same pathogen. The definition of an outbreak also depended on the specific pathogen found or the health complaints reported; these parameters were not specified beforehand for all possible infectious diseases. In general, we defined an outbreak as a higher incidence of an infectious disease in the study cohort than the expected incidence in the general Dutch population at the same time. This was assessed by a medical doctor with experience working in the field of infectious disease control.

### Data analysis

The primary outcome of this study was the association between potential risk factors during obstacle runs, demographics and the development of AGI, RI and SI. Attack rates were calculated for subgroups of exposure. To determine the association between different potential risk factors and development of infections, a univariable multilevel analysis was conducted. Multilevel analysis was performed to take into account the potential clustering of effects among the 17 runs and relative risks (RR) were calculated accordingly. Following the univariable analysis, we included factors associated with infection with a p value < 0.05 in univariable analysis in a multivariable multilevel binomial regression. We considered a p value of < 0.05 to be statistically significant. Data were analysed using SPSS statistics 21 (IBM, New York, United States (US)) and STATA 14 (StataCorp, College Station, Texas, US).

## Results

In total, 17 obstacle runs carried out over 14 weekends (between April and October 2017) in the Netherlands were included in this study. Of these runs, 12 were in the provinces of Noord-Brabant, two in Zuid-Holland, two in Gelderland and one in Limburg. The runs had ca 30,000 participants in total, ranging from 230–7,600 per run.

### Questionnaire response

Of the 30,000 participants, 2,900 started the questionnaire and 2,646 (91%) completed it. Distribution of the questionnaire was not consistent between the obstacle runs, as contact information (specifically email addresses) was not available for all participants, e.g. when one participant signed up on behalf of a group. Therefore, for nine obstacle runs the link to the questionnaire was posted on social media or the obstacle run’s website. Because of this, it was not possible to determine precisely how many participants were reached via email, social media or the website in order to calculate the response rate.

### Characteristics of the study population

The median age was 33 years (range: 5–71 years) and 1,435 (53%) were male. Following an obstacle run, 2.7% of respondents reported AGI, 3.7% reported SI and 5.8% reported RI (Table 1). The majority of respondents reported that they had no allergies or other chronic diseases (83.1% and 84.5%, respectively). A small number of respondents (8.1%) used medication at the time of the run.

**Table 1:** Characteristics of respondents from 17 obstacle runs, the Netherlands, 2017 (n = 2,900)

Characteristics	n	%
**Sex**	**2,693**	**100**
Male	1,435	53.3
Female	1,258	46.7
Missing	207	NA
**Age (years)**	**2,692**	**100**
< 18	311	11.6
19–25	363	13.5
26–35	863	32.1
36–45	784	29.1
> 45	371	13.8
Missing	208	NA
**Self-reported symptoms**		
**Acute gastrointestinal infections**	**2,808**	**100**
Yes	76	2.7
No	2,732	97.3
Missing	92	NA
**Skin infections**	**2,790**	**100**
Yes	103	3.7
No	2,687	96.3
Missing	110	NA
**Respiratory infections**	**2,790**	**100**
Yes	163	5.8
No	2,627	94.2
Missing	110	NA
**Current smoker**	**2,691**	**100**
Yes	185	6.9
No	2,506	93.1
Missing	209	NA
**Use of medication^a^**	**2,658**	**100**
Yes	216	8.1
No	2,442	91.9
Missing	242	NA
**Allergies^b^**	**2,658**	**100**
Yes	449	16.9
No	2,209	83.1
Missing	242	NA
**Other chronic diseases^c^**	**2,658**	**100**
Yes	411	15.5
No	2,247	84.5
Missing	242	NA
**Exposure to open water or obstacle run (past 3 months)**	**2,864**	**100**
Yes	1,080	37.7
No	1,784	62.3
Missing	36	NA

NA: not applicable.

^a^ For example, antibiotics or antacids.

^b^ For example, hay fever or other self-defined allergies.

^c^ Other than allergies; for example, diabetes, immune disorders or gastrointestinal diseases.

Additional information on the questions asked in the questionnaire can be found in Supplement S1.

### Characteristics of the obstacle runs

Table 2 describes the main characteristics assessed using the environmental checklist. Of 17 obstacle runs, 10 were single-day events, 13 took place on a day with no rain, 11 had running water for handwashing (but most did not have paper towels and soap), 16 had facilities for participants to rinse off afterwards and 14 handed out free food. At one obstacle run, fruit and packaged foods (i.e. granola bars) were distributed. At another, energy bars were handed out, but not fruit. Of the 13 obstacle runs where fruit was handed out, at six runs, volunteers peeled the fruit before distributing it to participants. Environmental samples were not tested, because there was no reported outbreak nor did respondents of the same run test positive for the same pathogen.

**Table 2:** Characteristics of the obstacle runs, the Netherlands, 2017 (n = 17)

Characteristics	n
**Number of participants**	
< 1,000	9
≥ 1,000	8
**Event duration (days)**	
1	10
2	7
**Weather conditions**	
Heavy rain	0
Light rain	4
No rain	13
**Temperature (°C)**	
< 15.0	0
15.1–20.0	7
20.1–25.0	6
> 25.1	4
**Swimming water**	
Only official	2
Only non-official	12
Both official and non-official^a^	3
**Hygiene facilities**	
Running water	11
Paper towels	4
Soap	4
Rinse facilities^b^	16
**Food and water distributed during obstacle run**	
Food	14
Fruit without peel^c^	6
Free drinking water	17
Animal faeces present on trail	7

^a^ Official swimming water was officially approved for swimming by local authorities and non-official swimming water was not checked by local authorities.

^b^ For example, shower, garden hose or cold water tub.

^c^ In 13 obstacle runs.

^d^ Depending on the obstacle run this was either bottled water or tap water.

Additional information on the questions asked in the questionnaire can be found in Supplement S1.

### Epidemiological determinants

#### Reported health complaints

In total, 641 of 2,813 (22.8%) respondents reported health complaints (e.g. headache, stomach ache and vomiting) following participation in an obstacle run. Of those, five discovered a tick during or after the run, 156 (5.6%) reported receiving a wound, 131 (4.7%) reported an injury (mostly concerning the knee (n = 45) and ankle (n = 25)), two respondents broke a bone and six tore a muscle.

In all three main health complaints reported, (AGI, RI and SI) females were more likely to report infections than males; AGI: 47 (68%) vs 22 (32%); SI: 68 (69%) vs 31 (31%); RI: 103 (69%) vs 47 (31%).

#### Determinants associated with acute gastrointestinal infections

Of 2,831 respondents, 38 had AGI during or in the week before the obstacle run. The multilevel univariable analysis showed that 10 determinants were associated with the development of AGI following an obstacle run. In the multivariable model, five remained statistically significant, including swallowing mud (RR: 2.4; 95% CI: 1.2–4.9), having allergies (RR: 1.7; 95% CI: 1.2–2.5) and being female (RR: 1.9; 95% CI: 1.2–2.8). Drinking alcohol on the day of an obstacle run and having chronic diseases other than allergies decreased the risk for AGI following an obstacle run (RR: 0.34; 95% CI: 0.16–0.71 and RR: 0.51; 95% CI: 0.33–0.79, respectively) (Table 3).

**Table 3:** Determinants for developing acute gastrointestinal infections in obstacle run participants, the Netherlands, 2017 (n = 2,808)

Variables	Total n^a^	Cases of AGI^b^	Attack rate (%)	Univariable analysis		Multivariable analysis	
				RR (95% CI)	p value	RR (95% CI)^c^	p value
**Respondent characteristics**							
**Age (years)**							
0–18	311	6	1.9	0.69 (0.22–2.2)	0.530	0.63 (0.21–1.9)	0.412
19–25	363	15	4.1	1.5 (0.71–3.1)	0.293	1.2 (0.55–2.6)	0.661
26–35	863	24	2.8	Ref		Ref	
36–45	784	22	2.8	1.01 (0.64–1.6)	0.969	1.01 (0.59–1.7)	0.964
> 45	371	2	0.54	0.19 (0.04–0.87)	**0.032**	0.23 (0.05–1.1)	0.067
**Sex**							
Male	1,435	22	1.5	Ref		Ref	
Female	1,258	47	3.7	2.4 (1.8–3.3)	**0.000**	1.9 (1.2–2.8)	**0.003**
**Current smoker**							
No	2,506	65	2.6	Ref		NA	
Yes	185	4	2.2	0.8 (0.35–2.0)	0.681	NA	
**Exposure to open water or obstacle run (past 3 months)**							
No	1,746	56	3.2	Ref		Ref	
Yes	1,062	20	1.9	0.59 (0.40–0.86)	**0.006**	0.71 (0.47–1.1)	0.118
**Use of medication (incl. antacids)^d^**							
No	2,442	62	2.5	Ref		NA	
Yes	216	6	2.8	1.09 (0.45–2.7)	0.843	NA	
**Use of antacids**							
No	2,637	67	2.5	Ref		NA	
Yes	21	1	4.8	1.9 (0.37–9.4)	0.444	NA	
**Allergies^e^**							
No	2,209	49	2.2	Ref		Ref	
Yes	449	19	4.2	1.9 (1.3–2.8)	**0.001**	1.7 (1.2–2.5)	**0.004**
**Chronic diseases other than allergies^f^**							
No	2,247	61	2.7	Ref		Ref	
Yes	411	7	1.7	0.63 (0.43–0.91)	**0.014**	0.51 (0.33–0.79)	**0.003**
**Run characteristics**							
**Distance (km)**							
0–4.9	190	3	1.6	0.54 (0.11–2.6)	0.446	NA	
5–7.9	1,093	32	2.9	Ref		NA	
8–10	469	12	2.6	0.87 (0.38–2.0)	0.750	NA	
10.1–15	786	21	2.7	0.91 (0.44–1.9)	0.808	NA	
> 15.1	270	8	3.0	1.01 (0.48–2.2)	0.975	NA	
**Outside temperature (°C)**							
≤ 15.0	0	0	NA	NA		NA	
15.1–20.0	1,149	40	3.5	Ref		NA	
20.1–25.0	802	17	2.1	0.61 (0.26–1.4)	0.255	NA	
≥ 25.1 °C	857	19	2.2	0.64 (0.30–1.4)	0.245	NA	
**Official swimming water^g^**							
Non-official	1,945	50	2.6	Ref		NA	
Official	411	11	2.7	1.04 (0.49–2.2)	0.918	NA	
Both	452	15	3.3	1.3 (0.70–2.4)	0.419	NA	
**Number of event days**							
1	1,201	22	1.8	Ref		NA	
2	1,607	54	3.4	1.8 (0.97–3.5)	0.064	NA	
**Animal faeces present on trail**							
No	1,856	58	3.1	Ref		NA	
Yes	952	18	1.9	0.61 (0.32–1.1)	0.122	NA	
**Specific exposure**							
**Type of shower water used**							
Tap water	2,364	59	2.5	Ref		NA	
Open water	236	7	3.0	1.2 (0.65–2.2)	0.573	NA	
Hot tub	80	2	2.5	1.002 (0.43–2.4)	0.997	NA	
Other	35	1	2.9	1.1 (0.37–3.5)	0.813	NA	
**Shower time (hours after run)**							
< 1	1,840	52	2.8	Ref		NA	
1–3	706	15	2.1	0.75 (0.43–1.3)	0.313	NA	
> 3	174	2	1.2	0.41 (0.17–0.95)	**0.037**	NA	
**Toilet used**							
No	862	18	2.1	Ref		NA	
Yes	1,856	51	2.8	1.3 (0.80–2.2)	0.276	NA	
**Water in mouth**							
No	1,092	16	1.5	Ref		Ref	
Yes, not swallowed	1,200	35	2.9	2.0 (1.1–3.8)	**0.036**	2.0 (0.94–4.1)	0.071
Yes, swallowed	440	19	4.3	2.9 (1.4–6.2)	**0.004**	2.2 (0.97–5.2)	0.061
**Mud in mouth**							
No	1,762	39	2.2	Ref		Ref	
Yes, not swallowed	882	24	2.7	1.2 (0.8–1.9)	0.350	1.02 (0.61–1.7)	0.946
Yes, swallowed	85	7	8.2	3.7 (1.8–7.7)	**0.000**	2.4 (1.2–4.9)	**0.015**
**Consumed beverages**							
**Drinking water from organisation**							
No	459	7	1.5	Ref		NA	
Yes	2,229	62	2.8	1.8 (0.79–4.2)	0.162	NA	
**Soda**							
No	2,130	60	2.8	Ref		NA	
Yes	558	9	1.6	0.57 (0.29–1.1)	0.105	NA	
**Energy drink**							
No	2,165	53	2.5	Ref		NA	
Yes	523	16	3.1	1.2 (0.73–2.1)	0.419	NA	
**Alcoholic beverage**							
No	2,031	62	3.1	Ref		Ref	
Yes	657	7	1.1	0.35 (0.18–0.67)	**0.002**	0.34 (0.16–0.71)	**0.004**
**Coffee/tea**							
No	2,196	55	2.5	Ref		NA	
Yes	492	14	2.9	1.1 (0.62–2.1)	0.682	NA	
**Juice**							
No	1,791	39	2.2	Ref		NA	
Yes	58	2	3.5	1.6 (0.27–9.4)	0.613	NA	

AGI: acute gastrointestinal infections; CI: confidence interval; incl.: including; NA: not applicable; Ref: reference; RR: relative risk.

^a^ Number of respondents who were exposed to the exposure variable.

^b^ Number of respondents with gastrointestinal infections in the week after the event and exposure to the exposure variable.

^c^ Adjusted for any exposure with p value < 0.05 in the univariable analysis.

^d^ For example, antibiotics or antacids.

^e^ For example, hay fever or other self-defined allergies.

^f^ Other than allergies; for example, diabetes, immune disorders and gastrointestinal diseases.

^g^ Official swimming water was officially approved for swimming by local authorities and non-official swimming water was not checked by local authorities.

Additional information on the questions asked in the questionnaire can be found in Supplement S1.

The questions on diarrhoea and vomiting were asked later on in the questionnaire and some respondents stopped before answering these questions. Only responses from those who answered these questions were included.

#### Determinants associated with skin infections

Five determinants were associated with the development of SI following an obstacle run in the univariable analysis and three remained statistically significant in the multivariable model. These included rinsing off after the run in a hot tub compared to running tap water and being female (RR: 2.2; 95% CI: 1.7–2.8 and RR: 2.3; 95% CI: 1.3–3.9, respectively). An outside temperature of > 25 °C decreased the risk for SI (RR: 0.53; 95% CI: 0.31–0.91) (Table 4).

**Table 4:** Determinants for developing skin infections in obstacle run participants, the Netherlands, 2017 (n = 2,790)

Variables	Total n^a^	Cases of SI^b^	Attack rate (%)	Univariable		Multivariable	
				RR (95% CI)	p value	RR (95% CI)^c^	p value
**Respondent characteristics**							
**Age (years)**							
0–18	311	11	3.5	0.92 (0.48–1.8)	0.819	1.2 (0.58–2.5)	0.607
19–25	363	21	5.8	1.5 (0.94–2.4)	0.089	1.4 (0.84–2.3)	0.202
26–35	863	33	3.8	Ref		Ref	
36–45	784	25	3.2	0.83 (0.46–1.5)	0.555	0.92 (0.50–1.7)	0.797
> 45	371	9	2.4	0.63 (0.38–1.1)	0.081	0.76 (0.42–1.4)	0.360
**Sex**							
Male	1,435	31	2.2	Ref		Ref	
Female	1,258	68	5.4	2.5 (1.4–4.6)	**0.003**	2.3 (1.3–3.9)	**0.004**
**Exposure to open water or obstacle run (past 3 months)**							
No	1,733	73	4.2	Ref		NA	
Yes	1,057	30	2.8	0.67 (0.39–1.2)	0.166	NA	
**Allergies^d^**							
No	2,209	76	3.4	Ref		NA	
Yes	449	22	4.9	1.4 (0.95–2.1)	0.087	NA	
**Chronic diseases other than allergies^e^**							
No	2,247	74	3.3	Ref		Ref	
Yes	411	24	5.8	1.8 (1.03–3.1)	**0.040**	1.7 (0.97–2.8)	0.065
**Run characteristics**							
**Outside temperature (°C)**							
≤ 15.0	0	0	NA	NA		NA	
15.1–20.0	1,137	62	5.5	Ref		Ref	
20.1–25.0	80	20	2.5	0.46 (0.23–0.89)	**0.022**	0.56 (0.31–1.005)	0.052
≥ 25.1 °C	853	21	2.5	0.45 (0.23–0.90)	**0.023**	0.53 (0.31–0.91)	**0.022**
**Number of event days**							
1	1,193	54	4.5	Ref		NA	
2	1,597	49	3.1	0.68 (0.30–1.5)	0.341	NA	
**Animal faeces present on trail**							
No	1,841	61	3.3	Ref		NA	
Yes	949	42	4.4	1.3 (0.64–2.8)	0.436	NA	
**Specific exposures**							
**Type of shower water used**							
Tap water	2,364	76	3.2	Ref		Ref	
Open water	236	16	6.8	2.1 (0.80–5.6)	0.133	1.5 (0.67–3.4)	0.319
Hot tub	80	6	7.5	2.3 (1.6–3.4)	**0.000**	2.2 (1.7–2.8)	**0.000**
Other	35	1	2.9	0.89 (0.10–8.1)	0.916	0.66 (0.08–5.4)	0.696
**Shower time (hours after run)**							
< 1	1,840	61	3.3	Ref		NA	
1–3	706	32	4.5	1.4 (0.96–2.0)	0.086	NA	
> 3	174	6	3.5	1.04 (0.50–2.1)	0.915	NA	
**Clothes worn**							
Long pants, long sleeves	333	14	4.2	Ref		NA	
Long pants, short sleeves/short pants, long sleeves combined	1,420	52	3.7	0.87 (0.49–1.6)	0.642	NA	
Short pants, short sleeves	889	30	3.4	0.80 (0.35–1.8)	0.603	NA	
Other	78	3	3.9	0.91 (0.29–2.9)	0.880	NA	

CI: confidence interval; NA: not applicable; Ref: reference; RR: relative risk; SI: skin infections.

^a^ Number of respondents who were exposed to the exposure variable.

^b^ Number of respondents with skin infections in the week after the event and exposure to the exposure variable.

^c^ Adjusted for any exposure with p value < 0.05 in the univariable analysis.

^d^ For example, hay fever or other self-defined allergies.

^e^ Other than allergies; for example, diabetes, immune disorders and gastrointestinal diseases.

Additional information on the questions asked in the questionnaire can be found in Supplement S1.

The question on skin infections was asked later on in the questionnaire and some respondents stopped before answering this question. Only responses from those who answered this question were included.

#### Determinants associated with respiratory infections

Five determinants were associated with the development of RI following an obstacle run in the univariable analysis and three remained statistically significant in the multivariable model. These included being aged 19–25 years (compared to 26–35 years) and being female (RR: 1.8; 95% CI: 1.2–2.9 and RR: 2.1; 95% CI: 1.6–3.0, respectively). Being aged ≥ 45 years (compared to 26–35 years) decreased the risk for developing RI (RR: 0.31; 95% CI: 0.16–0.60) (Table 5).

**Table 5:** Determinants for developing respiratory infections in obstacle run participants, the Netherlands, 2017 (n = 2,790)

Variables	Total n^a^	Cases of RI^b^	Attack rate (%)	Univariable		Multivariable	
				RR (95% CI)	p value	RR (95% CI)^c^	p value
**Respondent characteristics**							
**Age (years)**							
0–18	311	20	6.4	1.2 (0.88–1.7)	0.245	1.4 (0.98–2.0)	0.061
19–25	363	38	10	2.0 (1.2–3.1)	**0.003**	1.8 (1.2–2.9)	**0.011**
26–35	863	46	5.3	Ref		Ref	
36–45	784	41	5.2	0.98 (0.59–1.6)	0.941	1.1 (0.64–1.7)	0.842
> 45	371	5	1.4	0.25 (0.13–0.48)	**0.000**	0.31 (0.16–0.60)	**0.000**
**Sex**							
Male	1,435	47	3.3	Ref		Ref	
Female	1,258	103	8.2	2.5 (1.9–3.4)	**0.000**	2.1 (1.6–3.0)	**0.000**
**Current smoker**							
No	2,506	135	5.4	Ref		NA	
Yes	185	15	8.1	1.5 (0.92–2.5)	0.107	NA	
**Exposure to open water or obstacle run (past 3 months)**							
No	1733	109	6.3	Ref		NA	
Yes	1057	54	5.1	0.81 (0.56–1.2)	0.268	NA	
**Use of medication (incl. antacids)^d^**							
No	2,442	137	5.6	Ref		NA	
Yes	216	11	5.1	0.91 (0.52–1.6)	0.733	NA	
**Use of medication for allergies**							
No	2,604	142	5.5	Ref		NA	
Yes	54	6	11	2.0 (0.90–4.6)	0.087	NA	
**Use of medication for respiratory diseases**							
No	2,611	145	5.6	Ref		NA	
Yes	47	3	6.4	1.1 (0.42–3.1)	0.784	NA	
**Allergies^e^**							
No	2,209	109	4.9	Ref		Ref	
Yes	449	39	8.7	1.8 (1.1–2.8)	**0.014**	1.6 (0.99–2.6)	0.054
**Chronic diseases other than allergies^f^**							
No	2,247	116	5.2	Ref		NA	
Yes	411	32	7.8	1.5 (0.99–2.3)	0.054	NA	
**Run characteristics**							
**Outside temperature (°C)**							
≤ 15.0	0	0	NA	NA		NA	
15.1–20.0	1,137	78	6.9	Ref		NA	
20.1–25.0	800	50	6.3	0.91 (0.60–1.4)	0.667	NA	
≥ 25.1	853	35	4.1	0.60 (0.26–1.4)	0.235	NA	
**Rain on event day**							
Heavy rain	0	0	NA	NA		NA	
Light rain	663	41	6.2	Ref		NA	
No rain	2,127	122	5.7	0.93 (0.58–1.5)	0.750	NA	
**Number of event days**							
1	1,193	77	6.5	Ref		NA	
2	1,597	86	5.4	0.83 (0.51–1.4)	0.462	NA	
**Specific exposures**							
**Type of shower water used**							
Tap water	2,364	129	5.5	Ref		Ref	
Open water	236	18	7.6	1.4 (1.1–1.8)	**0.016**	1.3 (0.84–2.0)	0.246
Hot tub	80	4	5.0	0.92 (0.54–1.6)	0.749	1.2 (0.64–2.1)	0.626
Other	35	1	2.9	0.52 (0.05–5.2)	0.579	0.51 (0.06–4.3)	0.536
**Water in mouth**							
No	1,092	53	4.9	Ref		NA	
Yes, not swallowed	1,200	75	6.3	1.3 (0.84–2.0)	0.240	NA	
Yes, swallowed	440	25	5.7	1.2 (0.78–1.8)	0.451	NA	
**Mud in mouth**							
No	1,762	96	5.5	Ref		NA	
Yes, not swallowed	882	52	5.9	1.1 (0.74–1.6)	0.683	NA	
Yes, swallowed	85	5	5.9	1.1 (0.45–2.6)	0.863	NA	

CI: confidence interval; incl.: including; NA: not applicable; Ref: reference; RI: respiratory infections; RR: relative risk.

^a^ Number of respondents who were exposed to the exposure variable.

^b^ Number of respondents with respiratory infections in the week after the event and exposure to the exposure variable.

^c^ Adjusted for any exposure with p value < 0.05 in the univariable analysis.

^d^ For example, antibiotics or antacids.

^e^ For example, hay fever or other self-defined allergies.

^f^ Other than allergies; for example, diabetes, immune disorders or gastrointestinal diseases.

Additional information on the questions asked in the questionnaire can be found in Supplement S1.

The question on respiratory infections was asked later on in the questionnaire and some respondents stopped before answering this question. Only responses from those who answered this question were included.

### Clinical microbiological results

The laboratory received stool specimens from 111 respondents from 17 obstacle runs, of which 13 tested positive for six different pathogens (no participant tested positive for the same pathogen as another participant in the same run). These pathogens were sapovirus (n = 5), norovirus (n = 4), *Shigella* spp. (n = 1), enterohaemorrhagic *Escherichia coli* (EHEC) (n = 1), *Campylobacter jejuni* (n = 1) and *Giardia lamblia* (n = 2).

Four of these pathogens (norovirus, sapovirus, *G. lamblia* and *C. jejuni*) explained the acute gastrointestinal complaints of seven respondents, with two reporting gastrointestinal symptoms before the obstacle run and five after. The EHEC-positive respondent reported headache and red bumps on the skin. The other six positive respondents (*G. lamblia*, norovirus, sapovirus, *Shigella* spp.) did not report any health complaints.

## Discussion

To our knowledge, this is the first study investigating the incidence of AGI, SI and RI following participation in an obstacle run, as well as risk factors. Not many infectious diseases were reported by respondents in the questionnaire of this study (in 2.7%–5.8% of respondents), which suggests a low risk for infection after participation in an obstacle run.

The primary care continuous morbidity surveillance system estimated that from April to October 2017, 2% of the Dutch adult population had consulted a general practitioner (GP) for an AGI and 4% for an RI [21]; no primary care data was available for SI. Although the incidences of AGI and RI from our study are seemingly comparable to those obtained from primary care in the Netherlands, the latter only reflects diseases in people who attended a GP and, therefore, may not be generalisable to the general population.

In this study, the ingestion of mud was associated with AGI, supporting current advice offered to participants, i.e. to avoid ingesting water/mud by trying to keep their mouths closed during obstacle runs. This advice arose due to similar findings regarding the risk for infectious diseases observed in other (outbreak) investigations related to events that include water or mud, e.g. mountain bike events and city swims [7-14]. We acknowledge this is not always feasible due to the high oxygen demand during intense activity.

Previous studies have also found a protective effect of alcohol on the risk of developing AGI [22-25]. This effect could be attributed to the ethanol and antioxidants or other substances in the alcoholic beverages [25]. However, as we did not collect information on the number of alcoholic beverages consumed by participants, nor when they consumed it (i.e. before, during or after the obstacle run), the protective association we found should be interpreted with caution.

We found that respondents with allergies were more at risk for AGI in multivariable analysis and those with a chronic disease other than allergies were more at risk for SI and RI in univariable analysis. We also found that having one or more chronic diseases had a protective effect on AGI. This could partially be explained by the non-specific definition of chronic diseases in our study. Several chronic diseases (e.g. eczema and diabetes) were grouped together since the number of each reported disease was too low to analyse separately.

Proper handwashing is a very effective measure for the prevention of infectious disease [26]. In our study, we found that 13 obstacle runs did not have adequate handwashing facilities, e.g. with running water, soap and paper towels. Food was distributed at 14 obstacle runs and, as it is not practical for participants to wash their hands during an obstacle run (and they are likely covered in mud when the food is distributed), unpeeled fruits and packaged foods may be better options.

### Strengths and limitations

Due to the large study population and inclusion of several different obstacle runs, we believe the results could be relevant to other events with similar environmental conditions. Further, as several obstacle runs were investigated, the identified risk factors may be more generalisable.

There are several limitations with this study. First, it is likely that there was self-selection bias, wherein participants who developed symptoms after an obstacle run were more likely to take part in the study than those who remained healthy. This may have resulted in an overestimation of the attack rate for AGI, SI and RI.

Second, recall bias may have occurred, as participants received the questionnaire 1 week after participating in the run and exposure to potential risk factors may have been recalled better by respondents who developed symptoms. Although this bias is expected to be minimal, the RR and risk factors identified may have been overestimated.

Third, it is known that women tend to report poorer health than men on self-reported health indicators [27], which may explain the high incidence on AGI, SI and RI reported among women.

Fourth, the incidence of infectious diseases reported in this study may have been overestimated, as not all symptoms reported are exclusive to AGI, RI and SI. For example, respiratory disease can occur due to allergies. It was not possible, however, to differentiate between the underlying causes of certain symptoms, which could have resulted in an overestimation of the incidence of AGI, RI and SI in this study. Further, respondents were asked about infectious disease symptoms that occurred following the obstacle run, so the symptoms reported may not have been caused by the event.

Fifth, due to the design of the study, the results of the microbiological analysis were not compared with a control group. In future studies, however, stool sample testing should be done directly after onset of symptoms and a control group should be included so the results can be compared and used as part of an outbreak investigation, should the need arise.

Finally, we investigated frequencies of disease in this study, but diseases such as tularaemia and leptospirosis—which have longer incubation periods than the time given to complete the questionnaire—might have been missed. However, as these are rare diseases in the Netherlands and the symptoms are not widely recognised, it is unlikely that these diseases would have been identified even with a longer time period allocated to the questionnaire.

### Conclusion

Our study suggests that the risk of contracting AGI, SI or RI following participation in an obstacle run is low. However, the potential for disease outbreaks related to such events can be high, as seen in previous studies [7-14]. To limit the occurrence of outbreaks and sporadic infections, we recommend that organisers of obstacle runs inform participants of infectious disease risks and potential preventive measures they could take, e.g. practicing good hand hygiene, not participating if they are ill, not swallowing mud and showering directly after the run. In addition, we recommend that organisers adequately facilitate these preventive measures, e.g. by installing proper handwashing and shower facilities and only distributing foods that are unpeeled/packaged during the obstacle run. Based on visual inspections, we also recommend that organisers fulfil the national hygiene guidelines regarding the toilets and showers around the obstacle run course.

## Supplementary Data

140000 Supplementary Material S1

## References

[1] Obstakels.com. Wat is een obstacle run. Facts feiten. [What is an obstacle run. Facts]. Lieshout: Obstakels.com. [Accessed: 4 Sept 2019]. Dutch. Available from: https://obstakels.com/wat-is-een-obstacle-run/facts-feiten/<ref/

[2] Strong Viking. Legal. Policies and conditions. Website. [Accessed: 4 Sept 2019]. Dutch. Available from: https://strongviking.com/nl/condities/

[3] Obstakels.com. Obstacle Run Kalender. [Obstacle run calendar]. Lieshout: Obstakels.com. [Accessed: 29 Dec 2017]. Dutch. Available from: https://obstakels.com/kalender/

[4] Terrain Race. What is an obstacle course race? Website. [Accessed: 4 Sept 2019]. Available from: https://terrainrace.com/terrain-obstacle-course/

[5] Obstakels.com. Wat is nou een survivalrun? [What is a survival run?]. Lieshout: Obstakels.com. [Accessed 13 Aug 2018]. Dutch. Available from: https://obstakels.com/2015/11/24/wat-is-nou-een-survivalrun/

[6] All Terrain. What is a survival run? Website. [Accessed: 4 Sept 2019]. Available from: https://allterrain.nl/what-is-a-survivalrun/

[7] Griffiths SL Salmon RL Mason BW Elliott C Thomas DRH Davies C Using the internet for rapid investigation of an outbreak of diarrhoeal illness in mountain bikers. Epidemiol Infect. 2010;138(12):1704-11. 10.1017/S0950268810001561 20587125

[8] Hall V Taye A Walsh B Maguire H Dave J Wright A A large outbreak of gastrointestinal illness at an open-water swimming event in the River Thames, London. Epidemiol Infect. 2017;145(6):1246-55. 10.1017/S0950268816003393 28162113 PMC9507846

[9] Joosten R Sonder G Parkkali S Brandwagt D Fanoy E Mughini-Gras L Risk factors for gastroenteritis associated with canal swimming in two cities in the Netherlands during the summer of 2015: A prospective study. PLoS One. 2017;12(4):e0174732. 10.1371/journal.pone.0174732 28369101 PMC5378355

[10] Mexia R Vold L Heier BT Nygård K Gastrointestinal disease outbreaks in cycling events: are preventive measures effective? Epidemiol Infect. 2013;141(3):517-23. 10.1017/S0950268812000817 22591923 PMC9151891

[11] Six C Aboukais S Giron S D’Oliveira JC Peloux-Petiot F Franke F Outbreak of diarrhoeal illness in participants in an obstacle adventure race, Alpes-Maritimes, France, June 2015. Euro Surveill. 2016;21(23):30253. 10.2807/1560-7917.ES.2016.21.23.30253 27311488

[12] Stuart TL Sandhu J Stirling R Corder J Ellis A Misa P Campylobacteriosis outbreak associated with ingestion of mud during a mountain bike race. Epidemiol Infect. 2010;138(12):1695-703. 10.1017/S095026881000049X 20334726

[13] Zeigler M Claar C Rice D Davis J Frazier T Turner A Centers for Disease Control and Prevention (CDC) Outbreak of campylobacteriosis associated with a long-distance obstacle adventure race--Nevada, October 2012. MMWR Morb Mortal Wkly Rep. 2014;63(17):375-8. 24785983 PMC4584888

[14] Hintaran AD Kliffen SJ Lodder W Pijnacker R Brandwagt D van der Bij AK Infection risks of city canal swimming events in the Netherlands in 2016. PLoS One. 2018;13(7):e0200616. 10.1371/journal.pone.0200616 30052633 PMC6063404

[15] Zijlstra M Hulsker CCC Fanoy EB Pijnacker R Kraaijeveld A Koene MGJ [Tularaemia in a boy following participation in a mud race]. Ned Tijdschr Geneeskd. 2017;160(0):D1180. 28466800

[16] Uiterwijk M, De Rosa M, Friesema I, Valkenburgh S, Roest H, van Pelt W. Staat van Zoönosen 2015. [State of Zoonoses 2015]. Bilthoven: National Institute for Public Health and the Environment (RIVM); 2016. Dutch.

[17] De Schrijver K, Berghs R, Van Esbroeck M, Vlieghe E, Flipse W. Leptospirose bij deelnemers aan een obstakelloop in Nijlen in 2015. [Leptospirosis among participants in an obstacle course in Nijlen in 2015]. Vlaams infectieziektebulletin. 2017;2017(4):6-11.

[18] National Institute for Public Health and the Environment (RIVM). Gastroenteritis and food poisoning (foodborne). Bilthoven: RIVM; [Accessed 12 Sep 2019]. Dutch. Available from: https://lci.rivm.nl/draaiboeken/gastro-enteritis-en-voedselvergiftigingen

[19] National Institute for Public Health and the Environment (RIVM). Article 26 notifications Wpg settings. Bilthoven: RIVM; [Accessed 12 Sep 2019]. Dutch. Available from: https://lci.rivm.nl/draaiboeken/artikel-26-meldingen-wpg-instellingen

[20] Collector Innovative Surveys. Vragenlijsten [questionnaires]. Survalyzer AG [Accessed in 2017]. Dutch Available from: https://login.premiumuser.datacoll.nl/

[21] Hooiveld M, Donkers GA, Korevaar JC. Wekelijkse surveillance cijfers. Nivel Zorgregistraties eerste lijn. [Weekly surveillance figures. Nivel Primary Care Registration]. Utrecht: Nivel; 2018. Dutch. Available from: www.nivel.nl/surveillance.

[22] Bellido-Blasco JB Arnedo-Pena A Cordero-Cutillas E Canós-Cabedo M Herrero-Carot C Safont-Adsuara L The protective effect of alcoholic beverages on the occurrence of a Salmonella food-borne outbreak. Epidemiology. 2002;13(2):228-30. 10.1097/00001648-200203000-00020 11880766

[23] Correia AM Gonçalves G Gomes A Oliveira B Gonçalves J Miranda M The protective effect of alcoholic beverages in a foodborne outbreak of Salmonella Enteritidis PT1 in northern Portugal. Euro Surveill. 2003;7(13):2195.

[24] Desenclos JA Klontz KC Wilder MH Gunn RA The protective effect of alcohol on the occurrence of epidemic oyster-borne hepatitis A. Epidemiology. 1992;3(4):371-4. 10.1097/00001648-199207000-00013 1637901

[25] Romeo J Wärnberg J Nova E Díaz LE Gómez-Martinez S Marcos A Moderate alcohol consumption and the immune system: a review. Br J Nutr. 2007;98(S1) Suppl 1;S111-5. 10.1017/S0007114507838049 17922947

[26] World Health Organization (WHO). WHO guidelines on Hand Hygiene in Health Care: First Global Patient Safety Challenge Clean Care is Safer Care. Geneva: WHO; 2009. Available from: https://www.ncbi.nlm.nih.gov/books/NBK144004/ 23805438

[27] Boerma T Hosseinpoor AR Verdes E Chatterji S A global assessment of the gender gap in self-reported health with survey data from 59 countries. BMC Public Health. 2016;16(1):675. 10.1186/s12889-016-3352-y 27475755 PMC4967305

