# Supplementary material for: Risk factors for developing acute gastrointestinal, skin or respiratory infections following obstacle and mud run participation, the Netherlands, 2017
Source: Euro Surveill. 2019 Oct 3;24(40):1900088. doi: 10.2807/1560-7917.ES.2019.24.40.1900088 (PMC6784448; doi:10.2807/1560-7917.ES.2019.24.40.1900088)
Supplement: Supplementary Material S1 [file 1900088_BOOGERT_SupplementS1.pdf]

"This supplementary material is hosted by Eurosurveillance as supporting information alongside the article [Risk factors for developing infectious disease symptoms following obstacle and mud run participation in the Netherlands, 2017] on behalf of the authors who remain responsible for the accuracy and appropriateness of the content. The same standards for ethics, copyright, attributions and permissions as for the article apply. Supplements are not edited by Eurosurveillance and the journal is not responsible for the maintenance of any links or email addresses provided therein."

Municipal Health Service - Questionnaire mud- and obstacle run

Introduction

- Thanks for participating in our study!
- Even if you did not experience health complaints, it is important to complete the questionnaire
- You can complete the questionnaire before {DATE}
- Are you younger than 16 years old? Please fill in the questionnaire with a parent/guardian/mentor
- Questions relate to a two weeks period after the run (you do not have to wait for the period to be over to fill in the questionnaire)
- Information you enter will be anonymous
- Filling in the questionnaire will take approximately 5-10 minutes

1. What distance did you run? (multiple answers possible, in case of multiple distances ran, the rest of the questionnaire applies to the longest distance).
    a. 0-4.9 KM
    b. 5-7.9 KM
    c. 8-10 KM
    d. 10.1-15 KM
    e. 15.1+ KM
2. At what time did you approximately start with the run? Indicate time as follows: 12:10

………………………………………………….

3. How long did you take to complete the run (in minutes)?

………………………………………………….

4. Did you complete the run?
    a. Yes → Proceed to question 6
    b. No, after which obstacle did you stop? ………………………………….
5. Why did you stop?

…………………………………………………

6. Did you participate in another mud- or obstacle run, or have you swum in open water, in the past three months? If yes, please specify how many times approximately?
    a. Yes, namely ……
    b. No

The next part of the questionnaire relates to potential health complaints you might have experienced in the week prior to the run, between {DATE} and {DATE}.

7. Did you experience any of the following health complaints in the week prior to the run {DATE}?
    a. I did not experience any health complaints
    b. Nausea
    c. Vomiting
    d. Diarrhoea
    e. Fever
    f. Jaundice (yellow eyes and/or sudden yellow discoloration of the skin)
    g. Stomach ache
    h. Headache
    i. Red, irritated eyes
    j. Ear pain
    k. Cold, coughing or sore throat
    l. Red bumps or other skin abnormalities
    m. Musculoskeletal injury or fracture
    n. Muscle or joint pain
    o. Open wound (cuts and scrapes)
    p. Other, namely ……
8. Were these complaints over at the start of the run, {DATE}?
    a. Yes
    b. No, please specify which complaints were still present ………
    c. Not applicable, since I had no complaints

The next part of the questionnaire relates to possible experienced health complaints in the two weeks after the run, {DATE}.

9. Did you experience any health complaints in the two weeks following the run {DATE}. (For instance, gastro intestinal complaints, injuries, skin abnormalities or flu like complaints)
    a. Yes

b. No, proceed to question 64

10. Did you experience any nausea after the run?
    a. Yes
    b. No, proceed to question 13
11. When did these complaints start? *Answering options ranged from the date of the run until 14 days later*
12. How many days did you experience this complaint? *Answering options ranged from 'Still experiencing this complaint', 1 day to 14 days*
13. Did you experience any vomiting after the run?
    a. Yes
    b. No, proceed to question 16
14. When did these complaints start? *Answering options ranged from the date of the run until 14 days later*
15. How many days did you experience this complaint? *Answering options ranged from 'Still experiencing this complaint', 'Once', 1 day to 14 days.*
16. Did you experience any diarrhoea after the run?
    a. Yes
    b. No, proceed to question 20
17. When did these complaints start? *Answering options ranged from the date of the run until 14 days later*
18. How many days did you experience this complaint? *Answering options ranged from 'Still experiencing this complaint', 'Once', 1 day to 14 days*
19. What was the frequency of diarrhoea?
    a. 0-3 times a day
    b. 3-10 times a day
    c. More than 10 times a day
20. When did these complaints start? *Answering options ranged from the date of the run until 14 days later*
21. How many days did you experience this complaint? *Answering options ranged from 'Still experiencing this complaint', 1 day to 14 days*
22. Did you take your temperature, while you had a fever?
    a. No
    b. Yes, how high was your temperature? …………
23. Did you experience jaundice after the run? (Yellow eyes and/or sudden yellow discoloration of the skin)
    a. Yes

b. No, proceed to question 26

24. When did these complaints start? *Answering options ranged from the date of the run until 14 days later*
25. How many days did you experience this complaint? *Answering options ranged from 'Still experiencing this complaint', 1 day to 14 days*
26. Did you experience stomach ache after the run?
    a. Yes
    b. No, proceed to question 29
27. When did these complaints start? *Answering options ranged from the date of the run until 14 days later*
28. How many days did you experience this complaint? *Answering options ranged from 'Still experiencing this complaint', 1 day to 14 days*
29. Did you experience a headache after the run?
    a. Yes
    b. No, proceed to question 32
30. When did these complaints start? *Answering options ranged from the date of the run until 14 days later*
31. How many days did you experience this complaint? *Answering options ranged from 'Still experiencing this complaint', 1 day to 14 days*
32. Did you experience muscle or joint pain after the run (other or more severe than usually after this type of event)?
    a. Yes
    b. No, proceed to question 35
33. When did these complaints start? *Answering options ranged from the date of the run until 14 days later*
34. How many days did you experience this complaint? *Answering options ranged from 'Still experiencing this complaint', 1 day to 14 days*
35. Did you experience red or irritated eyes after the run?
    a. Yes
    b. No, proceed to question 38
36. When did these complaints start? *Answering options ranged from the date of the run until 14 days later*
37. How many days did you experience this complaint? *Answering options ranged from 'Still experiencing this complaint', 1 day to 14 days*
38. Did you experience ear pain after the run?
    a. Yes

b. No, proceed to question 41

39. When did these complaints start? *Answering options ranged from the date of the run until 14 days later*
40. How many days did you experience this complaint? *Answering options ranged from 'Still experiencing this complaint', 1 day to 14 days*
41. Did you experience a cold, coughing or sore throat after the run?
    a. Yes
    b. No, proceed to question 44
42. When did these complaints start? *Answering options ranged from the date of the run until 14 days later*
43. How many days did you experience this complaint? *Answering options ranged from 'Still experiencing this complaint', 1 day to 14 days*
44. Did you experience red bumps or other skin abnormalities after the run?
    a. Yes
    b. No, proceed to question 47
45. When did these complaints start? *Answering options ranged from the date of the run until 14 days later*
46. How many days did you experience this complaint? *Answering options ranged from 'Still experiencing this complaint', 1 day to 14 days*
47. Did you develop a muscle or joint injuries or fracture during the run?
    a. Yes, namely ………………
    b. No, proceed to question 49
48. At which obstacle did you sustain this injury or fracture? (Give a description of the obstacle)

………………………………………………………

49. Did you contract an open wound during the run?
    a. Yes
    b. No, proceed to question 51
50. At which obstacle did you contract this open wound? (give a description of the obstacle)

…………………………………………………………

51. Did you contract a tick bite (or tick) during the run?
    a. Yes
    b. No
52. Did you experience any other health complaints after the run?
    a. Yes, namely ……………………
    b. No, proceed to question 55

53. When did these complaints start? *Answering options ranged from the date of the run until 14 days later*
54. How many days did you experience this complaint? *Answering options ranged from 'Still experiencing this complaint', 1 day to 14 days*
55. Did you seek medical attention for your complaints? (either at the day of the run or after the run)
    a. Yes
    b. No, proceed to question 61
56. What form of medical attention did you seek? (multiple answers possible)
    a. First aid at the event
    b. General practitioner
    c. First aid at the hospital
    d. Admitted to the hospital
    e. Other, namely ………………
57. For which complaint did you seek help?

………………………………………………………

58. Were any samples taken for further analysis (for example, faeces, blood urine nasal or throat swabs or wound fluid)?
    a. Yes
    b. No, proceed to question 61
59. What kind of material was taken and send for further analysis?
    a. Blood
    b. Faeces
    c. Urine
    d. Throat swab
    e. Nasal swab
    f. Wound fluid
    g. Other, namely ……………………
60. What is the result of the laboratory analysis?

……………………………………………………………

61. If you experienced any health complaints, what do you think the explanation is for your complaints?

……………………………………………………………

62. In the week prior to your health complaints, were there any other persons (in your surrounding) experiencing similar complaints?
    a. Yes, they participated in the run
    b. Yes, they were at the run as spectator

c. Yes, they were not present at the run
d. No

The next part of the questionnaire relates to possible exposures and lifestyle factors that could have influenced your health.

63. Did you possibly get any water from the trail in your mouth?
    a. Yes
    b. No, proceed to question 67
64. Did you swallow this water?
    a. Yes
    b. No, proceed to question 67
65. How many times did you swallow the water?
    a. Less than 3 sips
    b. Between 3 and 6 sips
    c. More than 6 sips
66. Did you possibly get any mud from the trail in your mouth?
    a. Yes
    b. No, proceed to question 70
67. Did you swallow this mud?
    a. Yes
    b. No, proceed to question 70
68. How many times did you swallow the mud?
    a. Less than 3 times
    b. Between 3 and 6 times
    c. More than 6 times
69. What kind of cloths did you wear during the run?
    a. Long pants, long sleeves
    b. Long pants, short sleeves
    c. Short pants, long sleeves
    d. Short pants, short sleeves
    e. Other, namely ………………
70. Where did you first rinse of after the run?
    a. Showers at the event
    b. Showers or bath at home
    c. Other, namely ………………

71. In what time after the run did you rinse of?
    a. Within 1 hour after the run
    b. Between 1 and 3 hours after the run
    c. More than 3 hours after the run
72. Did you use (mobile) toilets or urinals at the event?
    a. Yes
    b. No, proceed to question 75
73. If you have used (mobile) toilets or urinals, when did you use them? (multiple answers possible)
    a. Morning
    b. Afternoon
    c. Evening
    d. Night
74. Did you see someone vomit during the event?
    a. Yes, where and when? ……………………
    b. No
75. Did get or buy something to eat at the event?
    a. Yes, handed out by the organisation, namely (e.g. banana) ………..
    b. Yes, bought at the event, namely (e.g. French fries) ……………………
    c. No
76. Did you drink any of the following drinks on the day of the run? (multiple answers possible)
    a. Yes, water handed out by the organisation
    b. Yes, soft drinks
    c. Yes, energy drink
    d. Yes, alcoholic beverages
    e. Yes, coffee/tea
    f. Yes, smoothie/jus d'orange
    g. No, none of the above

Personal characteristics

77. What is your gender?
    a. Male
    b. Female
78. What is your age (in years)?

    ……………………………………………

79. What is your length (in centimetres)?

………………………………………………

80. What is your weight (in kilograms)?

………………………………………………

To find out whether certain lifestyle factors might be related to developing health complaints, we will now ask a few questions about this.

81. How many times a week did you work out in the past three months (on average)?
    a. Less than once a week
    b. 1-2 times a week
    c. 3 or more times a week
82. Do you smoke?
    a. Yes
    b. No, proceed to question 85
83. How many cigarettes do you smoke?
    a. Less than one cigarette a day
    b. 1 to 5 cigarettes a day
    c. 5 or more cigarettes a day

The next part of the questionnaire will be about your general health.

84. Did you receive a tetanus- or DTP vaccination in the past 10 years?
    a. Yes
    b. No
    c. Not sure
85. At the moment, do you have one or more of the following chronic diseases? (multiple answers possible)
    a. Diabetes
    b. Hay fever or another allergy, namely ………………………
    c. Eczema
    d. Another skin condition than eczema, namely ……………………
    e. Absence of the spleen
    f. A liver disease, namely …………………………
    g. A kidney disease, namely …………………………
    h. Cardiovascular disease, namely ………………………
    i. Leukaemia or another type of cancer, namely …………………

j. An immune disorder, namely …………………..
k. Pulmonary disease, namely …………………..
l. A gastrointestinal disease, namely ………………..
m. Rheumatic arthritis (rheumatism
n. I have had a transplantation of ……………..
o. I have been treated with immunoglobulin or had a blood transfusion in the past three months
p. Other, namely …………………..
q. No, none of the above

86. At the time of the run, did you use any medication, for instance antacids or antibiotics?
    a. Yes, namely …………………..
    b. No

The answers from this questionnaire will be analysed anonymously. You can choose to leave your contact details to have a chance at winning a T-shirt. In some cases (e.g. when some answers are unclear) the Municipal Health Service might contact you for clarification.

87. Do you agree to leave your contact details, so the Municipal Health Service van contact you if there are any questions about your entry? By leaving your contact details, you also have a chance at winning a T-shirt.
    a. Yes
    b. No, proceed to question 92
88. What is your phone number and email address? (These contact details will be used for this research only, and will be deleted after)
    a. Phone number ………………………….
    b. Email address ………………………….
89. Surname and initials

………………………………………………..

91. What are the four digits of your postal code?

………………………….

92. Do you have any comments? (optional)

…………………………………………………………………………………….

Many thanks for filling out the questionnaire! The winner of the T-shirt will be contacted personally.

Closing page

Many thanks for your participation in this survey.

- Do you become ill in the next few weeks? Please contact our Municipal Health Service via *{e-mail}* or *{phone number}.*

We hope your participation will give us better insights in the risk factors for participants of mud- and obstacle runs. This will keep the runs fun and even healthier for everyone.

If you have any questions about the research, please contact the Municipal Health Service via *{e-mail}* or *{phone number}.*
